# Skin-to-skin contact and breastfeeding practices in Nigeria: a study of socioeconomic inequalities

**DOI:** 10.1186/s13006-021-00444-7

**Published:** 2022-01-03

**Authors:** Michael Ekholuenetale, Amadou Barrow, Amit Arora

**Affiliations:** 1 Department of Epidemiology and Medical Statistics, Faculty of Public Health, College of Medicine, University of Ibadan, Ibadan, Nigeria; 2 Department of Public & Environmental Health, School of Medicine & Allied Health Sciences, University of The Gambia, Kanifing, The Gambia; 3 School of Health Sciences, Western Sydney University, Campbelltown Campus, Locked Bag 1797, Penrith, NSW 2751 Australia; 4 Health Equity Laboratory, Campbelltown, NSW 2560 Australia; 5 Translational Health Research Institute, Western Sydney University, Locked Bag 1797, Penrith, NSW 2751 Australia; 6 Discipline of Child and Adolescent Health, Sydney Medical School, Faculty of Medicine and Health, The University of Sydney, Westmead, NSW 2145 Australia; 7 Oral Health Services, Sydney Local Health District and Sydney Dental Hospital, NSW Health, Surry Hills, NSW 2010 Australia

**Keywords:** Breastfeeding, Early initiation of breastfeeding, Infant feeding, Skin-to-skin contact (SSC), Socioeconomic inequalities

## Abstract

**Background:**

The effects of breastfeeding practices on children’s health are undoubtedly of great interest. However, inequalities in breastfeeding practices and mother and newborn skin-to-skin contact (SSC) exist in many resource-constrained settings. This study examined the regional prevalence and socioeconomic inequalities in exclusive breastfeeding (EBF), early initiation of breastfeeding and SSC in Nigeria.

**Methods:**

Data on 2936 infants under six months were extracted from the 2018 Nigeria Demographic and Health Survey (NDHS) to determine EBF. In addition, data on 21,569 children were analysed for early initiation of breastfeeding and SSC. Concentration index and curves were used to measure socioeconomic inequalities in EBF, early initiation of breastfeeding and SSC.

**Results:**

The prevalence of EBF, early initiation of breastfeeding and SSC were 31.8, 44.2 and 12.1% respectively. Furthermore, Ogun state had the highest prevalence of EBF (71.4%); while Bayelsa state had the highest prevalence of SSC (67.8%) and early initiation of breastfeeding (96.2%) respectively. Urban dwellers had higher prevalence of EBF, SSC and early initiation of breastfeeding across household wealth quintile and by levels of mothers’ education in contrast to their rural counterparts. We quantified inequalities in early initiation of breastfeeding, EBF, and SSC according to household wealth and maternal education. The study outcomes had greater coverage in higher household wealth, in contrast to the lower household wealth groups; early initiation of breastfeeding (concentration index = 0.103; *p* = 0.002), EBF (concentration index = 0.118; *p* < 0.001), and SSC (concentration index = 0.152; *p* < 0.001) respectively. Furthermore, early initiation of breastfeeding (concentration index = 0.091; *p* < 0.001), EBF (concentration index = 0.157; *p* < 0.001) and SSC (concentration index = 0.156; *p* < 0.001) had greater coverage among mothers with higher educational attainment.

**Conclusion:**

Low prevalence and socioeconomic inequalities in early initiation of breastfeeding, EBF and SSC were identified. We recommend that health promotion programs targeted and co-designed with disadvantaged mothers are critical to meet global breastfeeding targets. Also, future researchers should conduct further studies especially clinical control trials and qualitative studies to unravel the possible reasons for differences in the indicators.

## Background

Optimal breastfeeding practices have large recognition for health benefits [1, 2]. World Health Organization (WHO) recommends early initiation of breastfeeding for newborn within the first hour of birth [3]. This is also referred to as timely breastfeeding. It is recommended that infants be exclusively breastfed for the first six months of life [2]. On the other hand, exclusive breastfeeding (EBF) is the practice whereby an infant receives only breast milk for the first six months, with no other liquids or solid food, except oral rehydration solutions, drops and syrups [3, 4].

Early initiation of breastfeeding is associated with mother and newborn skin-to-skin contact (SSC), which reduces the risk of hypothermia [5], and strengthens maternal bonding with the infant [6]. The findings from a previous intervention study led to the development of guidelines that allowed early initiation of breastfeeding and SSC be included in the policy of newborn care [7]. In recent years, efforts have been made to improve newborn and infant feeding practices, including International Code of Marketing of Breast-milk Substitutes [8], Global Strategy for Infant and Young Child Feeding (IYCF) [3] and Baby Friendly Hospital Initiative (BFHI) [9]. WHO has provided a set of indicators to evaluate infant and young child feeding and determine the progress of breastfeeding advancement efforts. As a result, there has been an increased attention on infant and young child feeding pattern to ascertain optimal breastfeeding practices [2].

The WHO defined SSC as when a newborn is placed on the mother’s abdomen or chest in direct ventral-to-ventral skin-to-skin contact [10]. Early SSC is defined as beginning from any time after delivery to 23 h after birth and should be uninterrupted for at least 60 min [10]. SSC improves the newborn’s maintenance of blood glucose levels, temperature regulation and metabolic adaptation [11]. At birth, the newborn has a reduced capacity to generate heat due to metabolic activities. It is against this backdrop that maintenance of temperature is required for newborn at delivery. During SSC, there is a transfer of heat from the mother to her child, wherewith the mother’s body temperature activates the child’s sensory nerves, which in turn results in the child’s relaxation and reduces the tone of the sympathetic nerves [11].

Approximately 37% of children under six months are exclusively breastfed in resource-constrained settings [12]. Sub-optimal breastfeeding has been reported to be responsible for 1.4 million childhood deaths and about 10% of total disease burden among under 5 children [13]. Optimal breastfeeding practices are crucial in improving the health of children, and are associated with reduction in the risk of childhood morbidity and mortality [3, 14]. Early initiation of breastfeeding can reduce neonatal mortality [15]. Albeit, the prevalence of early initiation of breastfeeding is only about 50% in many resource-constrained settings [15]. The guideline for breastfeeding practices include the initiation of breastfeeding for all newborns within the first hour of life, while EBF is practiced for infants less than 6 months [2, 16]. Interestingly, SSC plays a mediating role in early initiation of breastfeeding [5].

High prevalence of hypothermia is recorded in settings with large number of newborn deaths, where hypothermia has become a major concern to improve the survival of newborn [17]. There is need for thermal care as newborns are commonly susceptible to hypothermia without prejudice to tropical climates. Newborns have thin skin, a large body surface area, little insulating fat and easily overwhelmed thermoregulatory mechanisms [18]. In the absence of thermal protection, newborns are unable to maintain body temperature, while preterm babies become most-at-risk of the adverse effects [19]. Several estimates of hypothermia in African settings are limited to hospital studies and ranged between 44 and 85% [20]. Besides the benefits to the newborn, SSC has been linked with many benefits for mothers. For instance, secretion of oxytocin in mothers who practice SSC strengthens uterine contractions, which aids the placenta to separate and the duration of the third stage of labour is shortened [21]. As a simple and cost-effective mechanism, SSC is recommended to improve post-delivery care and potentially save the lives of newborns and mothers [22]. Though WHO recommends SSC, the separation of mothers and newborns exists in many health facilities where newborns are often placed under warmers or in cots [23].

Evidence-based studies have reported that EBF and early initiation of breastfeeding are associated with large gains and improve childhood survival, as well as support the recommendations to start breastfeeding immediately after childbirth [24]. Despite the numerous advantages of optimal breastfeeding practices [25], the rates of EBF and early initiation of breastfeeding in several resource-constrained settings remains worrisome. Using nationally representative data, previous studies reported a low EBF prevalence of 16.4% [4], while another study reported EBF prevalence of 14% and early initiation of breastfeeding prevalence of 38% [26] and 34.7% [27] respectively. Moreover, only 10% of newborns in Nigeria received SSC [28]. These reports are worrisome and an urgent call to improve infant health care is required.

Breastfeeding practices and SSC benefit newborns in many ways, by providing warmth and nutrients to facilitate growth and enhance immunity [5, 29]. Understanding the patterns of breastfeeding practices is essential to prioritise filling the knowledge gaps for childhood survival [30]. Nonetheless, there are disparities in the coverage of infant feeding practices across geographical regions, household wealth status and parental educational level. Studies have reported that women from higher family income households, those with higher levels of education or those with partners having higher education or executive occupations were more likely to have optimal breastfeeding practices, when compared with those from low family income households, with no formal education, those whose partners have no formal education or unemployed respectively [26, 31]. Women with no formal education or low household wealth index have poor breastfeeding indicators, when compared to women with some level of education [32, 33]. Similarly, a previous study reported disparities in early initiation of breastfeeding across geographical regions in Nigeria, as North-Central region had the highest early initiation of breastfeeding rates [34] compared to other regions. To the best of our knowledge, there is no study in Nigeria that has examined regional prevalence or wealth-related and education inequalities in EBF, early initiation of breastfeeding and SSC using socioeconomic analytical tools. In light of the above, this study aims to examine the regional prevalence and socioeconomic inequalities in early initiation of breastfeeding, EBF, and SSC in Nigeria.

## Methods

### Data sources

We analysed a cross-sectional data extracted from Nigeria Demographic and Health Survey (NDHS) 2018. MEASURE DHS provided technical input in the process of data collection and is supported by the National Population Commission (NPC). NDHS is a vital source of data on EBF, early initiation of breastfeeding and SSC especially as it consists of a nationally representative sample of households. Demographic and Health Survey (DHS) data was collected through a stratified multistage cluster sampling technique. The procedure for stratification approach divides the population into groups by geographical region and commonly crossed by place of residence – urban versus rural. A multi-level stratification approach was used to divide the population into first-level strata and then subdivide the first-level strata into second-level strata, and so on. A two-level stratification in DHS is region and urban/rural stratification. DHS data is available in the public domain and accessed upon approval from DHS. The details of DHS data has been reported in a previous study [35]. Data on 2936 children under six months was extracted for the EBF analysis. In addition, data on 21,569 children was analysed for early initiation of breastfeeding and SSC respectively.

### Response rate

NDHS 2018 selected a total of 41,668 households for the sample, of which 40,666 were occupied. Of the occupied households, 40,427 were successfully interviewed, yielding a response rate of 99%. In the households interviewed, 42,121 women aged 15–49 were identified for individual interviews. Interviews were completed with 41,821 women, yielding a response rate of 99% [36].

### Selection and measurement of variables

**Outcomes**

- Early initiation of breastfeeding: In this study, early initiation of breastfeeding was defined as children who were put to breast within 1 h of birth. We coded as “1” if a child was put to breast early and “0” if a child was put to breast after 1 h of birth.
- Exclusive breastfeeding (EBF): We defined EBF as infants aged 0–6 months who were fed exclusively with breast milk. We coded as “1” if a child was exclusively breastfed and “0” if otherwise.
- Skin-to-skin contact (SSC): We defined SSC as when a child was put on mother’s chest and bare skin immediately after birth. We coded as “1” if a child had SSC and “0” if otherwise.

### Explanatory factors

Women’s educational attainment was categorised as: no formal education, primary school, secondary school, and higher education.

Household wealth quintile was computed by DHS using principal components analysis (PCA) to assign the wealth indicator weights. In their computation, they assigned scores and standardised the wealth indicator variable using household assets including; wall, floor, roof and wall type; whether a household had improved versus unimproved sanitation amenities and water source; whether a household had essential assets such as electricity, radio, television, cooking fuel, refrigerator, furniture amongst others. Furthermore, the factor loadings and z-scores were calculated. For each household, they multiplied the indicator values by factor loadings and summed to produce the household’s wealth index value. The standardised z-score was disentangled to classify the overall scores to wealth quintiles; poorest, poorer, middle, richer and richest [37]. Household wealth quintiles and mothers’ educational attainment were used as measures of socioeconomic status similar to previous studies [38].

Residential status was classified as urban versus rural.

Geographical region and states were measured as:

- North Central: Benue, Federal Capital Territory, Kogi, Kwara, Nasarawa, Niger, Plateau;
- North East: Adamawa, Bauchi, Borno, Gombe, Taraba, Yobe; North West: Jigawa, Kaduna, Kano, Katsina, Kebbi, Sokoto,Zamfara;
- South East: Abia, Anambra, Ebonyi, Enugu, Imo
- South South: Akwa-Ibom, Bayelsa, Cross River, Edo, Delta, Rivers;
- South West: Ekiti, Lagos, Ogun, Ondo, Osun, Oyo.

### Ethical consideration

This study was based on an analysis of population-based data that exist in public domain and available online with all identifier information removed. The authors were granted access to use the data by MEASURE DHS/ICF International. DHS Program is consistent with the standards for ensuring the protection of respondents’ privacy. ICF International ensures that the survey complies with the U.S. Department of Health and Human Services regulations for the respect of human subjects. The DHS project sought and obtained the required ethical approval from the National Health Research Ethics Committee (NHREC) in Nigeria before the surveys were conducted. No further consent was required for this study.

### Statistical analysis

Stata Version 14 (StataCorp., College Station, TX, USA) was used for data analysis. Stata survey module (‘svy’) was used with adjustment for the sample design. Percentage and Chi-square tests were used for summary statistics and bivariate analysis respectively. To determine socioeconomic inequalities in EBF, early initiation of breastfeeding and SSC, we used concentration index and present it graphically with the concentration curve. When the concentration index value is positive or the curve lies below the diagonal line (line of equality), it indicates that EBF, early initiation of breastfeeding and SSC coverage is greater among high socioeconomic groups. Conversely, when concentration index value is negative or the curve is above the line of equality, it indicates that EBF, early initiation of breastfeeding and SSC coverage is higher among low socioeconomic groups. The concentration index was used to decipher socioeconomic inequalities using Erreygers adjustment. The statistical significance was determined at *p* < 0.05.

## Results

We estimated the prevalence of EBF (31.8%), early initiation of breastfeeding (44.2%) and SSC (12.1%) respectively.

Table 1 shows that Benue (65.3%), Ondo and Osun (66.7%), Ekiti (69.6%), and Ogun (71.4%) had the leading prevalence of EBF respectively. Furthermore, Oyo (45.4%) and Bayelsa (67.8%) reported the highest prevalence of SSC in Nigeria. In addition, Niger (74.9%), Kogi (78.4%), Ogun (78.6%) and Bayelsa (96.2%) reported the highest prevalence of early initiation of breastfeeding respectively. Similar differences were obtained across geographical zones.

**Table 1:** Summary statistics from 36 States + Federal Capital Territory on exclusive breastfeeding, early initiation of breastfeeding and skin-to-skin contact between mother and newborn; NDHS, 2018

State	Exclusive breastfeeding		Skin-to-skin contact		Early initiation of breastfeeding	
	n	%	n	%	n	%
**North Central**						
Benue	72	65.3	599	20.2	591	64.3
Federal Capital Territory	71	46.5	519	6.7	529	64.7
Kogi	53	18.9	411	8.0	402	78.4
Kwara	54	51.9	466	5.2	456	61.6
Nasarawa	72	48.6	537	13.0	527	23.5
Niger	120	12.5	786	3.7	754	74.9
Plateau	63	46.0	521	1.7	515	52.4
Total estimate	505	39.0	3839	8.4	3774	60.3
*P*		< 0.001*		< 0.001*		< 0.001*
**North East**						
Adamawa	86	16.3	620	32.7	606	20.3
Bauchi	129	24.8	893	15.1	882	14.0
Borno	67	7.5	670	6.4	663	51.3
Gombe	124	30.7	816	22.9	798	20.7
Taraba	104	30.8	709	2.5	698	11.6
Yobe	101	40.6	766	9.1	734	51.8
Total estimate	611	26.5	4474	14.7	4381	27.7
*P*		< 0.001*		< 0.001*		< 0.001*
**North West**						
Jigawa	118	9.3	901	37.6	879	11.3
Kaduna	147	19.7	895	6.4	866	34.5
Kano	168	12.5	1247	8.2	1210	40.4
Katsina	146	25.3	929	17.1	914	39.9
Kebbi	96	14.6	824	5.5	794	28.2
Sokoto	104	20.2	703	0.4	672	14.3
Zamfara	128	39.8	804	2.5	790	52.9
Total estimate	907	20.3	6303	11.5	6125	32.5
*P*		< 0.001*		< 0.001*		< 0.001*
**South East**						
Abia	55	21.8	386	6.5	387	47.8
Anambra	93	34.4	536	9.0	537	39.3
Ebonyi	93	47.3	609	7.6	603	
Enugu	49	24.5	360	2.2	349	47.0
Imo	52	15.4	438	2.7	422	47.4
Total estimate	342	31.6	2329	6.0	2298	43.4
*P*		< 0.001*		< 0.001*		< 0.001*
**South South**						
Akwa-Ibom	49	30.6	377	13.8	365	57.3
Bayelsa	45	35.6	373	67.8	366	96.2
Cross River	36	41.7	307	11.7	301	65.1
Edo	45	37.8	306	7.5	297	63.0
Delta	40	25.0	328	8.5	335	44.8
Rivers	48	33.3	423	9.5	435	52.6
Total estimate	263	33.8	2114	20.4	2099	63.0
*P*		< 0.001*		< 0.001*		< 0.001*
**South West**						
Ekiti	46	69.6	353	8.8	345	52.2
Lagos	59	59.3	555	8.7	577	57.7
Ogun	42	71.4	364	0.8	359	78.6
Ondo	54	66.7	400	7.3	391	48.1
Osun	39	66.7	371	5.7	366	64.5
Oyo	68	51.5	467	45.4	453	73.3
Total estimate	308	63.0	2510	13.7	2491	62.3
*P*		< 0.001*		< 0.001*		< 0.001*

*Significant at *P* < 0.05; *P* value was obtained using Chi-square test

Table 2 presents the percentages of EBF, SSC and early initiation of breastfeeding across household wealth quintile and maternal education. Urban dwellers had higher EBF, SSC and early initiation of breastfeeding across household wealth quintile and levels of mother’s education, in contrast to their rural counterparts. The concentration index quantified the degree of wealth-related and maternal education inequalities in EBF, SSC and early initiation of breastfeeding. Overall, the study outcomes had greater coverage in households with higher wealth, in contrast to households with lower wealth; EBF (concentration index = 0.118; *p* < 0.001), SSC (concentration index = 0.152; *p* < 0.001) and early initiation of breastfeeding (concentration index = 0.103; *p* = 0.002) respectively. Furthermore, EBF had greater coverage among mothers with higher educational attainment, compared with children from mothers with lower educational attainment (concentration index = 0.157; *p* < 0.001), SSC (concentration index = 0.156; *p* < 0.001) and early initiation of breastfeeding (concentration index = 0.091; *p* < 0.001). In addition, the concentration indices between urban and rural residence were compared to determine if there were differences. Based on the results, statistical significance were obtained in EBF, SSC and early initiation of breastfeeding for maternal educational attainment. However, for household wealth, the difference between urban and rural residence was only statistically significant for early initiation of breastfeeding.

**Table 2:** Prevalence and concentration index of exclusive breastfeeding, early initiation of breastfeeding and skin-to-skin contact between mother and newborn in Nigeria

	Exclusive breastfeeding			Skin-to-skin contact			Early initiation of breastfeeding		
	Urban	Rural	Total	Urban	Rural	Total	Urban	Rural	Total
**Household wealth quintile**									
Poorest (%)	51.1	24.8	26.6	5.2	8.1	7.9	46.7	31.5	32.8
Poorer (%)	28.0	23.7	24.3	9.4	10.7	10.5	45.0	38.2	39.2
Middle (%)	37.4	26.8	30.7	12.5	12.5	12.5	46.5	48.8	47.9
Richer (%)	37.6	41.7	39.3	15.4	13.4	14.6	49.7	53.5	51.2
Richest (%)	47.9	34.8	45.0	18.0	17.4	17.9	55.5	55.0	55.4
Overall (%)	40.4	27.4	31.8	14.5	10.8	12.1	50.3	40.8	44.2
**Concentration index**	0.061	0.083	0.118	0.125	0.121	0.152	0.042	0.114	0.103
Standard error	0.021	0.021	0.015	0.015	0.013	0.010	0.006	0.006	0.004
*P*-value^α^	0.004*	< 0.001*	< 0.001*	< 0.001*	< 0.001*	< 0.001*	< 0.001*	< 0.001*	< 0.001*
**Rural-urban comparison**									
z-stat	0.73			−0.18			8.55		
Difference in concentration index values	0.022			−0.004			0.073		
*P*-value^β^	0.464			0.855			< 0.001*		
**Mother’s education**									
No formal education (%)	30.7	21.3	22.9	11.6	8.2	8.8	43.7	34.3	35.9
Primary (%)	32.0	27.0	28.6	12.3	11.4	11.8	50.2	44.5	46.5
Secondary (%)	45.8	37.2	41.5	14.2	15.6	14.9	51.7	51.7	51.7
Higher (%)	45.8	52.5	48.1	21.7	17.1	20.4	54.9	51.8	54.0
Overall (%)	40.4	27.4	31.8	14.5	10.8	12.1	50.3	40.8	44.2
**Concentration index**	0.086	0.149	0.157	0.109	0.157	0.156	0.037	0.097	0.091
Standard error	0.021	0.019	0.014	0.015	0.012	0.010	0.006	0.005	0.004
*P*-value^α^	< 0.001*	< 0.001*	< 0.001*	< 0.001*	< 0.001*	< 0.001*	< 0.001*	< 0.001*	< 0.001*
**Rural-urban comparison**									
z-stat	2.26			2.46			7.43		
Difference in concentration index values	0.063			0.048			0.060		
*P*-value^β^	0.033*			0.014*			< 0.001*		

*Significant at *p* < 0.05; SE standard error; *P*-value^α^ and *P*-value^β^ were obtained using Concentration Index for overall inequalities across socioeconomic groups and measuring rural versus urban differences, respectively

**Fig. 1:**
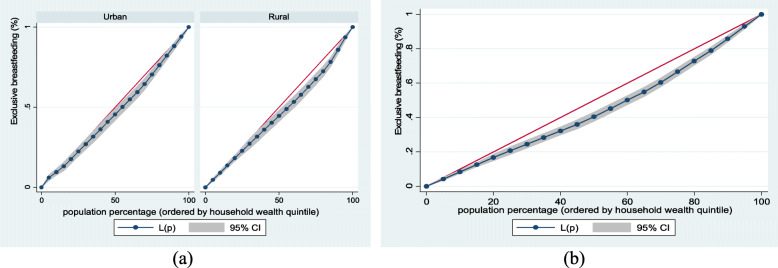
Exclusive breastfeeding by household wealth level (**a**) urban-rural and (**b**) overall

**Fig. 2:**
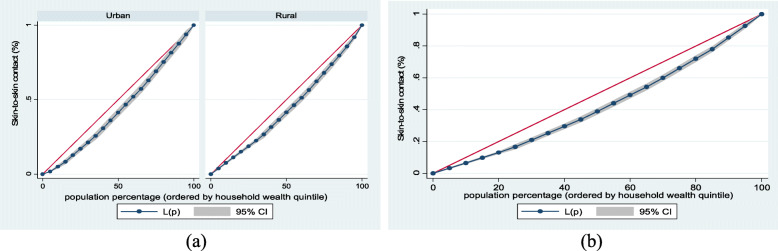
Mother and newborn skin-to-skin contact by household wealth level (**a**) urban-rural and (**b**) overall

**Fig. 3:**
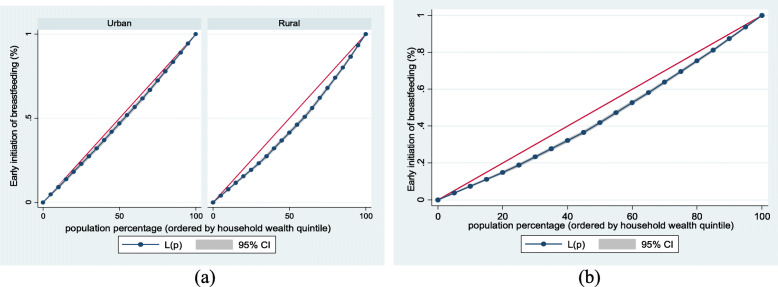
Early initiation of breastfeeding by household wealth level (**a**) urban-rural and (**b**) overall

Figure 1 (exclusive breastfeeding by household wealth level (a) urban-rural and (b) overall), Fig. 2 (mother and newborn skin-to-skin contact by household wealth level (a) urban-rural and (b) overall) and Fig. 3 (early initiation of breastfeeding by household wealth level (a) urban-rural and (b) overall) show the household wealth inequalities for EBF, SSC and early initiation of breastfeeding. The greater the degree of inequality, the more the curves sag away from the axis of equality. The areas between the curve and the line of inequality were maximal among rural children for EBF, SSC, and early initiation of breastfeeding, indicating that differences in household wealth level were greater. This is in line with the findings of the concentration index model.

**Fig. 4:**
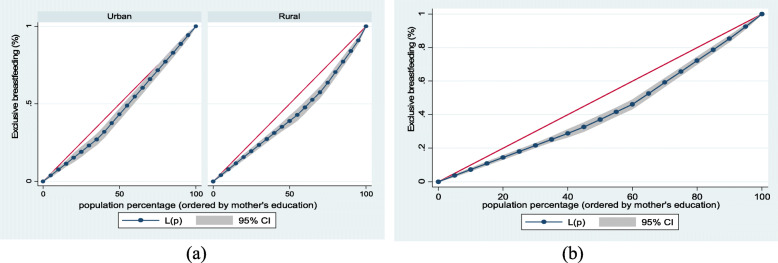
Exclusive breastfeeding by mothers’ education (**a**) urban-rural and (**b**) overall

**Fig. 5:**
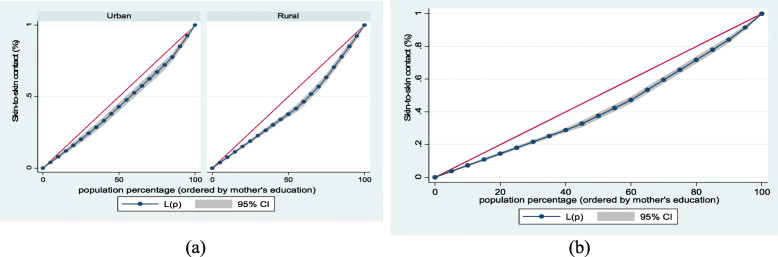
Mother and newborn skin-to-skin contact by mothers’ education (**a**) urban-rural and (**b**) overall

**Fig. 6:**
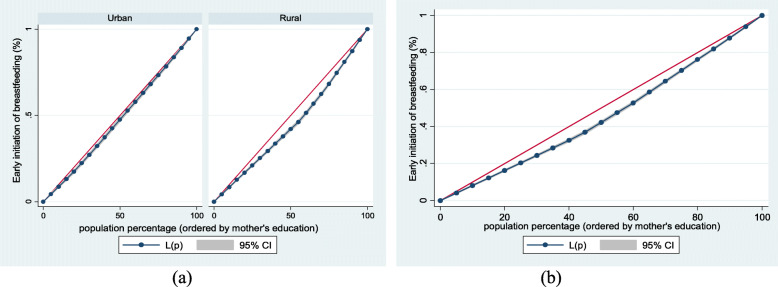
Early initiation of breastfeeding by mothers’ education (**a**) urban-rural and (**b**) overall

Figure 4 (exclusive breastfeeding by mothers’ education (a) urban-rural and (b) overall), Fig. 5 (mother and newborn skin-to-skin contact by mothers’ education (a) urban-rural and (b) overall) and Fig. 6 (early initiation of breastfeeding by mothers’ education (a) urban-rural and (b) overall) show mother’s educational attainment inequalities for EBF, SSC and early initiation of breastfeeding. The greater the degree of inequality, the more the curves deviate from the line of equality. As the areas between the curve and the line of disparity were maximal, disparities in mothers’ educational achievement differed among children based on breastfeeding practices and SSC. This clearly showed that those with higher educational attainment had higher EBF, SSC, and early initiation of breastfeeding.

## Discussion

The findings from this study bring to limelight the prevalence and socioeconomic differences in EBF, early initiation of breastfeeding and SSC which need urgent attention in Nigeria. Ogun state had the highest prevalence of EBF (71.4%); while Bayelsa state had the highest prevalence of SSC (67.8%) and early initiation of breastfeeding (96.2%) respectively. Urban dwellers had higher prevalence of early initiation of breastfeeding, EBF, and SSC across household wealth quintile and by levels of mothers’ education in contrast to their rural counterparts. The study outcomes had greater prevalence in higher household wealth and among mothers with higher educational attainment.

Despite the known benefits of breastfeeding, the findings of this study showed that less than half of women (44.2%) and about one-thirds (31.8%) initiated breastfeeding in the first hour of birth and practiced EBF respectively. However, these data show some improvement over results of previous studies in Nigeria. For example, using the 2003 data, EBF rates of about 16.4% was reported in Nigeria [4], and in another study using 2008 data reported early initiation of breastfeeding rate of 38% and EBF rate of 14% [26]. In the results from 2013 data, the proportion of infants who initiated breastfeeding within the first hour of birth was 34.7% [27]. These rates including the findings of this study highlight significant gaps in achieving the target of 90% of women to exclusively breastfeed their infants in the first 6 months of life - a practice that is associated with 10% reduction of under-5 deaths [39]. The low prevalence among educated women may be attributed to early resumption for those who work in the private sector. Though some governments have approved six months maternity leave for mothers in their employment, however, for mothers in the private sector, they do not have that privilege.

The impact of SSC in providing an appropriate and affordable childhood care is well known. Moreover, it can easily be practiced, even in primary health care centres and has the potential to save newborns’ and mothers’ lives amongst other benefits [5]. Albeit, just over one-tenth of women (12.1%) practiced SSC in Nigeria in the present study. This finding is similar to a previous report of 10% SSC prevalence in Nigeria [28]. The practice of SSC in other African countries varies widely. In Gambia, the prevalence of SSC was reported to be 35.7% [40] whereas SSC is rarely practiced in Tanzania (< 1%) [41], Uganda and Mali (both approximately 2%) [42]. In Ethiopia, approximately 9 to13% mother and newborn SSC were reported in previous studies [43, 44] and the prevalence of SSC in Ghana is reported to be approximately 10% [45]. These results are concerning and highlight the low prevalence of SSC in Sub-Saharan African countries given the benefits of SSC in thermoregulation and prevention of newborn deaths [17].

In a previous study in African countries including Nigeria, it has been reported that maternal beliefs such as *vernix caseosa* was related to poor maternal behaviours which acts as a significant barrier to practice SSC. Based on the findings, early bathing of the newborn is a common practice in Nigeria due to a deep-rooted belief that delay in bathing the newborn may result in body odour. It is concerning that when asked about keeping the baby warm, respondents across study sites rarely mentioned the recommended thermal care practices (SSC), suggesting that these were not perceived as salient [46]. Such beliefs and norms may be responsible for low prevalence of SSC in the general population.

In this study, there was a social gradient reported in study outcomes. A higher socioeconomic status would help to improve early initiation of breastfeeding, EBF and SSC by enhancing accessibility to health information which could positively influence health care seeking behaviours [47]. In this study, we found that women who had formal education or those in higher household wealth had higher coverage of early initiation of breastfeeding, EBF and SSC. These findings are similar to previous studies which report formal maternal education to significantly improve SSC and early initiation of breastfeeding [7, 32, 33]. A simple and cost effective educational intervention achieved the inclusion of SSC and early initiation of breastfeeding as part of standard maternal health care continuum [7]. In this study, mothers from higher socio-economic class had higher coverage of EBF in contrast to their disadvantaged counterparts. This is in agreement with previous studies which found an association between household wealth, maternal education and breastfeeding, as only one-tenth of mothers who practiced EBF came from poor households and without formal education, in contrast to their counterparts [4, 29, 48–50]. It is worth noting women from higher socioeconomic status have higher levels of health literacy and have easier access to resources and act more positively to health promotion messages.

The improvement in breastfeeding practices among educated mothers, indicates the substantial impact of mother’s education on infant health, well-being and development. This is consistent with the findings from previous studies whereby elementary education became the basic threshold needed to gain health information, as well as provided women, specifically the disadvantaged, with self-confidence and the autonomy required to act appropriately. Conversely, women with no formal education are known to have poor knowledge and attitude towards optimal breastfeeding practices. It is recommended frequent contact of women and their partners with healthcare providers may enhance information on optimal breastfeeding practices [51]. Therefore, stakeholders in public health are obliged to design interventions or policies to aid disadvantaged mothers, for example those with poor or no formal education to access health facility for information (for example, during antenatal visit) to promote optimal breastfeeding practices in Nigeria.

### Strength and limitation

The present study uses a nationally representative data covering a large sample size from the 2018 NDHS to reach plausible conclusions on breastfeeding practices and SSC. Furthermore, this study has become the foremost to examine socioeconomic inequalities in early initiation of breastfeeding, EBF and SSC using vital socioeconomic tools. The results from this study fill the knowledge gap for socioeconomic inequalities in early initiation of breastfeeding, EBF and SSC. Nonetheless, there is a potential for recall bias which could lead to overestimation or underestimation of the outcome variables. Mothers were asked how their child was fed in the preceding 24 h and their responses may not be completely accurate. Furthermore, the definition of EBF was based on a 24-h recall, and the day-to-day variability might lead to recall or measurement bias. In addition, DHS does not collect data on household income or expenditure, which are the traditional indicators to measure wealth status. The assets-based wealth index was used as a proxy indicator for household socioeconomic status which may not be complete accurate representation of socio-economic status.

## Conclusion

There was low prevalence in early initiation of breastfeeding, EBF and SSC. Moreover, these practices were influenced by mother’s educational attainment and household wealth quintiles. Notably, women with formal education and those with higher household wealth quintile had higher rates of early initiation of breastfeeding, EBF and SSC compared to their counterparts. There is an urgent need to address socioeconomic inequalities in early initiation of breastfeeding, EBF and SSC. Health promotion programs targeted and co-designed with disadvantaged mothers in Nigeria are critical to meet global breastfeeding targets. We recommend future researchers to conduct further studies especially clinical control trials and qualitative studies to unravel the possible reasons for regional differences in the indicators (early initiation of breastfeeding, EBF and SSC).

## Data Availability

Data for this study were sourced from Demographic and Health surveys (DHS) and available here: http://dhsprogram.com/data/available-datasets.cfm.

## Abbreviations

BFHI: Baby friendly hospital initiative
EBF: Exclusive breastfeeding
ICF: Inner city fund
IYCF: Infant and young child feeding
NDHS: Nigeria demographic and health survey
NHREC: National health research ethics committee
NPC: National population commission
PCA: Principal components analysis
SE: Standard error
SSC: Mother and newborn skin-to-skin contact
WHO: World Health Organization
